# Differential Expression of Long Non-Coding RNA IGF2-AS in Tamoxifen-Resistant Breast Cancer Cells

**DOI:** 10.3390/biomedicines13092087

**Published:** 2025-08-27

**Authors:** Jeeyeon Lee, Byeongju Kang, Eun Ae Kim, Jieun Kang, Yee Soo Chae, Ho Yong Park, Soo Jung Lee, In Hee Lee, Ji-Young Park, Nora Jee-Young Park, Jin Hyang Jung

**Affiliations:** 1 Department of Surgery, School of Medicine, Kyungpook National University, Daegu 41944, Republic of Korea; j.lee@knu.ac.kr (J.L.); libertas033@knu.ac.kr (B.K.); phy123@knu.ac.kr (H.Y.P.); 2 Kyungpook National University Chilgok Hospital, Daegu 41404, Republic of Korea; yschae@knu.ac.kr (Y.S.C.); sj.lee@knu.ac.kr (S.J.L.); ihleeoncology@knu.ac.kr (I.H.L.); jyparkmd@knu.ac.kr (J.-Y.P.); pathpjy@knu.ac.kr (N.J.-Y.P.); 3 Exosome Convergence Research Center, Kyungpook National University, Daegu 41944, Republic of Korea; eunaenim1@knu.ac.kr (E.A.K.); cronin10@knu.ac.kr (J.K.); 4 Department of Oncology, School of Medicine, Kyungpook National University, Daegu 41944, Republic of Korea; 5 Department of Pathology, School of Medicine, Kyungpook National University, Daegu 41944, Republic of Korea

**Keywords:** breast cancer, tamoxifen, resistance, lncRNA, IGF2-AS

## Abstract

**Background**: Breast cancer, particularly the luminal subtype, often responds to endocrine therapies. However, 20–30% of patients develop resistance, resulting in more aggressive disease. Long non-coding RNAs (lncRNAs) are implicated in cancer progression and treatment resistance. **Objective**: This study aimed to evaluate the role of the lncRNA insulin-like growth factor 2 antisense (IGF2-AS) in tamoxifen-resistant breast cancer and assess its potential as a therapeutic target. **Methods**: Two tamoxifen-resistant breast cancer cell lines (TAMR-V and TAMR-H) were used to assess IGF2-AS expression via qPCR. Knockdown experiments with siRNA evaluated the role of IGF2-AS in cell proliferation, invasion, and migration. Next-generation sequencing (NGS) analyzed gene expression differences between the cell lines. Kaplan–Meier survival analysis determined the clinical significance of IGF2-AS expression in breast cancer patients. **Results**: IGF2-AS expression was significantly upregulated in TAMR-V and TAMR-H cell lines compared to control MCF-7 cells. Knockdown of IGF2-AS reduced cell proliferation and invasion in TAMR-V cells but did not significantly affect TAMR-H cells, indicating a cell line-specific role in tamoxifen resistance. NGS revealed differential gene expression profiles between TAMR-V and TAMR-H cells, suggesting variability in resistance mechanisms. Survival analysis demonstrated that higher IGF2-AS expression was associated with poorer prognosis in breast cancer patients, including those with hormone-positive and triple-negative subtypes. **Conclusions**: IGF2-AS is upregulated in tamoxifen-resistant breast cancer and promotes cell proliferation and invasion in a cell line-specific manner. Its differential expression in TAMR-V and TAMR-H cells highlights the complexity of resistance mechanisms, suggesting IGF2-AS as a potential therapeutic target for overcoming tamoxifen resistance.

## 1. Introduction

Breast cancer is the most commonly diagnosed malignancy worldwide in females, with an estimated 2.2 million new cases and 0.6 million deaths reported in 2022, according to the GLOBOCAN database [1]. It accounts for a significant proportion of cancer-related morbidity and mortality, particularly among women.

Breast cancer is a highly heterogeneous disease, and it can be classified into molecular subtypes based on the expression of key receptors: estrogen receptor (ER), progesterone receptor (PR), and human epidermal growth factor receptor 2 (HER2) [2,3,4]. The main subtypes are luminal, HER2-positive, and triple-negative breast cancer (TNBC). The luminal subtype, characterized by hormone receptor (HR)-positive and HER2-negative status, accounts for approximately 65–75% of all breast cancer cases and is associated with a better prognosis [5,6]. However, resistance to tamoxifen remains a major clinical obstacle, often leading to recurrence and poor prognosis. Therefore, understanding the molecular basis of tamoxifen resistance and identifying novel therapeutic targets is critical.

Endocrine treatments, including tamoxifen, aromatase inhibitors, and selective estrogen receptor modulators (SERMs), are primary treatments for HR-positive breast cancer. However, 20–30% of patients develop resistance to endocrine therapy, leading to more aggressive disease [7,8]. Researchers are focusing on molecular studies to understand resistance mechanisms to hormone therapy and develop treatments to overcome or prevent this resistance [9,10,11,12].

Long non-coding RNAs (lncRNAs) are reported to be upregulated or downregulated in various malignancies and have critical roles in regulating genomic functions [13,14,15]. Several lncRNAs, including HOTAIR, H19, DSCAM-AS1, UCA1, and ROR, are implicated in hormone-resistant breast cancer [16,17,18,19,20,21]. The novel lncRNA, insulin growth factor 2 antisense (IGF2-AS), is also associated with breast cancer [22].

Overexpression of IGF2 in MCF-7 breast cancer cells has been linked to phenotypic changes, driving breast cancer progression [23]. IGF2-AS, transcribed in the opposite direction to IGF2, was first observed through its elevated expression in Wilms’ tumor [24]. Given its regulatory capabilities, it is hypothesized that this noncoding RNA may influence cell growth and development by modulating IGF2 expression [25,26,27]. This suggests that IGF2-AS could counteract IGF2, potentially regulating cancer cell proliferation and hormone resistance mechanisms.

Previous studies showed higher IGF2-AS expression in tamoxifen-resistant breast cancer cell lines compared to other subtypes. Therefore, this study aimed to explore the role of IGF2-AS in tamoxifen-resistant breast cancer and assess its potential as a therapeutic target to overcome tamoxifen resistance.

## 2. Materials and Methods

### 2.1. Cell Culture and Tissue Samples of Human Breast Cancer

MCF-7 breast cancer cells (HR-positive luminal-A sort) were obtained from the American Type Culture Collection (ATCC, Manassas, VA, USA). These cells were created in Dulbecco’s Modified Eagle’s Medium (DMEM; Gibco, Breathtaking Island, NY, USA) supplemented with 10% fetal bovine serum (Gibco).

Two sorts of tamoxifen-resistant MCF-7 breast cancer (TAMR) cell lines were utilized: (1) TAMR cells were saved in DMEM with 10% FBS and 7–10 mol/L tamoxifen (Sigma-Aldrich, St. Louis, MO, USA), provided by Dr. Santen, University of Virginia Health Sciences System (TAMR-V cells) [28,29]; (2) the TAMR cells were saved in DMEM with 10% FBS containing 10% FBS, 10 µg/mL human insulin (Sigma-Aldrich; MO, USA), and 1 µM 4-hydroxytamoxifen (4-OH, Sigma-Aldrich; MO, USA), obtained from ATCC (CRL-3435, Manassas, VA, USA) in 2020 (TAMR-H cells) [30].

Patient tissue samples were obtained following written informed consent from all the participants, in accordance with institutional ethical standards. This study was approved by the Institutional Review Board (IRB) of Kyungpook National University Chilgok Hospital, Daegu, Republic of Korea (KNUCH 2015-05-205) on 16 December 2022.

### 2.2. RNA Extraction, LncRNA Profiling, and Quantitative RT-PCR

Total RNA was isolated from the breast cancer cells utilizing RNAiso Plus (Takara, Otsu, Japan). Following the manufacturer’s instructions, a reverse transcription polymerase chain reaction (RT-PCR) was performed with SuperScript III Reverse Transcriptase (Invitrogen, Carlsbad, CA, USA). RNA concentration was measured utilizing the NanoDrop ND-2000 (Thermo Scientific, Wilmington, DE, USA). For lncRNA expression profiling, 2 µg of RNA was reverse-transcribed to cDNA utilizing Human LncProfilersTM qPCR Array Kits (System Biosciences, Mountain View, CA, USA), according to the manufacturer’s recommendations.

IGF2-AS expression levels were quantified by RT-PCR utilizing TaqMan Gene Expression Master Mix (Applied Biosystems, Foster City, CA, USA). TaqMan probes for IGF2-AS (Hs00212651_m1) and β-actin (Hs99999903_m1) were purchased from Thermo Fisher Scientific (Thermo Fisher Scientific, Waltham, MA, USA). The mRNA expression levels were normalized to β-actin utilizing the 2^−ΔΔCt^ methodology, and each test was analyzed in triplicate.

### 2.3. Transfection

Small interfering RNA (siRNA) for IGF2-AS was obtained from Thermo Fisher Scientific (Rockford, IL, USA). Transfection of si-Control or si-IGF2-AS into cells was performed utilizing Lipofectamine RNAiMAX (Thermo Fisher Scientific), based on the protocol of the manufacturer. After 48 h of transfection, total RNA was extracted, and real-time RT-PCR was chosen to assess the silencing effect.

### 2.4. MTT Assay

TAMR-V and TAMR-H cells were inoculated into a total volume of 200 µL in a 96-well plate, with 6 × 10^3^ cells per well. At each time point, 50 µL of MTT (2 mg/mL) was added to the wells, and the cells were incubated at 37 °C for 4 h. Then, 150 μL of dimethyl sulfoxide was added after removing the medium, and the mixture was shaken for 10 min. The quantity of formazan at a level presumed to be proportional to the number of viable cells was measured by recording absorbance changes at 570 nm using a microplate spectrophotometer (Agilent, Winooski, VT, USA). All the tests were conducted in triplicate.

### 2.5. Cell Invasion Assay

Transwell chambers with 8 μm pore inserts were coated with Matrigel (Corning, Tewksbury, MA, USA) and solidified by overnight incubation at 37 °C. After transfection, the cells were resuspended in DMEM containing 1% FBS and seeded on the upper side of the chamber at a thickness of 1 × 104 cells. Medium supplemented with 10% FBS was added to the lower part of the chamber. After 48 h of culture, cells on the interior surface of the chamber bottom were cleared with a cotton swab, fixed for 5 min with 2% paraformaldehyde, stained for 15 min with 0.5% crystal violet, and washed with phosphate-buffered saline and distilled water. The assaulted cells in four random fields were counted under a light microscope (Olympus CKX 53; Olympus Co., Tokyo, Japan). The microscope magnification was 200×.

### 2.6. Wound Healing Assay

TAMR-V and TAMR-H cells were seeded in six-well culture plates and cultured overnight to allow monolayer formation. A scratch was then created across the cell monolayer using a sterile pipette tip, followed by a medium change. The cells were subsequently transfected with 25 pmol of scrambled negative control siRNA or siRNA against IGF2-AS and incubated for 72 h. The wound area was monitored and quantified at designated time points using the ImageJ tool. Wound area quantification was performed using ImageJ version 1.54 g with the “MRI Wound Healing Tool” plugin. The wound area measured at 0 h was used as the baseline for comparison. For the scratch assay, 200 μL pipette tips were used to generate wounds in the cell monolayer prior to transfection. Images were captured at 0, 48, and 72 h using an Olympus CKX53 inverted microscope (Olympus Corporation, Tokyo, Japan) at 40× magnification. Data analysis was conducted using Microsoft Excel.

### 2.7. Next-Generation Sequencing (NGS)

Libraries were prepared from total RNA utilizing the NEBNext Ultra II Directional RNA Library Prep Kit for Illumina (New England BioLabs, Inc., Ipswich, MA, USA). mRNAs were detached by using a Poly(A) RNA Selection Kit (LEXOGEN, Inc., Vienna, Austria). At that point, isolated mRNAs were utilized for cDNA synthesis and shearing, following the manufacturer’s instructions. Requesting was performed utilizing Illumina records 1–12, and improvement was completed through PCR amplification. Library quality was assessed by utilizing the Agilent 2100 Bioanalyzer (DNA High Sensitivity Kit, Agilent) for evaluation of the mean fragment size and then assessed using the Library Quantification Kit on a StepOne Real-Time PCR System (Life Developments Inc., Florissant, MO, USA). High-throughput paired-end sequencing (100 bp) was performed on the Illumina NovaSeq 6000 platform (Illumina Inc., San Diego, CA, USA).

Raw sequencing data underwent quality control using FastQC [31]. Adapter sequences and low-quality reads (Q < 20) were removed using FASTX_Trimmer [32] and BBMap [33]. Then, the trimmed reads were mapped to the reference genome using TopHat [34]. Gene expression levels were assessed using fragments per kilobase of transcript per million mapped reads (FPKM) through Cufflinks [35]. FPKM values were normalized using the quantile normalization method within EdgeR [36], in the R programming environment [37].

Data analysis and graphical visualization were performed using ExDEGA (E-biogen Inc., Seoul, Republic of Korea).

### 2.8. Prognosis Estimation and Statistical Analysis

To evaluate the biological function of IGF-AS2 in breast cancer, we conducted a survival analysis using the web-based Kaplan–Meier (KM) plotter tool (available at http://kmplot.com). Overall survival (OS) was analyzed in 2976 breast cancer patients, regardless of endocrine treatment or chemotherapy. Additionally, OS was assessed in 2302 breast cancer patients who received endocrine treatment and in 126 patients with triple-negative breast cancer.

Data from three or more independent experiments are presented as means (±standard deviation, SD). We used Excel for statistical analysis, using data from three or more independent experiments presented as means (±standard deviation, SD). Differences between the two groups were evaluated for statistical significance using Student’s *t*-test, with statistical significance defined as *p* < 0.05, *p* < 0.01, or *p* < 0.001.

## 3. Results

### 3.1. IGF2-AS Was Significantly Upregulated in TAMR Cell Lines

Using qPCR array analyses, we screened for lncRNAs associated with tamoxifen-resistant breast cancer cells (TAMR-V and TAMR-H). Among 14 lncRNAs, we extracted transcripts that were either upregulated (expression change > 2-fold) or downregulated (expression change < 0.5-fold) compared to MCF-7. Whereas anti-NOS2A, EVF1 and EVF2, GAS5-family, H19 upstream conserved 1 & 2, HAR1B, and IGF2-AS were upregulated, 7SK, NTT, and SOX2OT were downregulated (Table 1).

**Figure 1:**
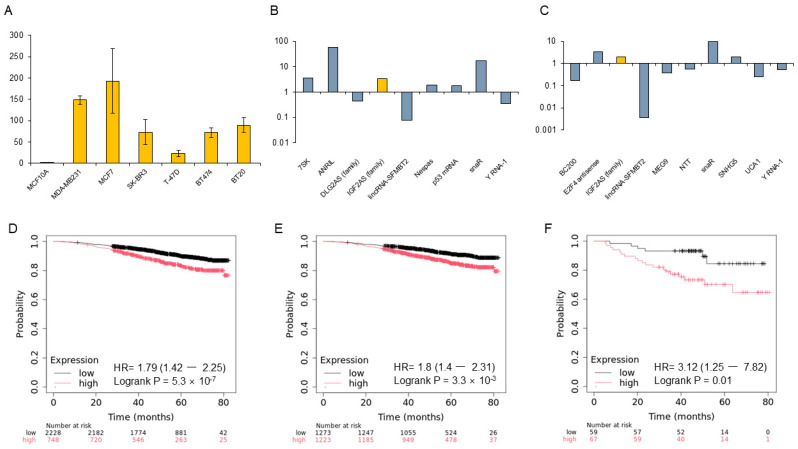
Expression of IGF2-AS in breast cancer cell lines and prognosis in the Kaplan–Meier (KM) plotter database. (**A**) Expression levels of IGF2-AS in various breast cell lines. Yellow bars represent mean IGF2-AS expression levels, and error bars indicate standard deviation (SD) from three independent experiments. (**B**,**C**) Upregulation of IGF2-AS in MCF7 and MDA-MB231 cell lines. (**D**) Overall prognosis based on IGF2-AS expression levels in breast cancer. (**E**,**F**) High IGF2-AS expression is associated with significantly poorer prognosis in hormone-positive breast cancers treated with endocrine therapy and in triple-negative breast cancers, according to the KM plotter database.

**Figure 2:**
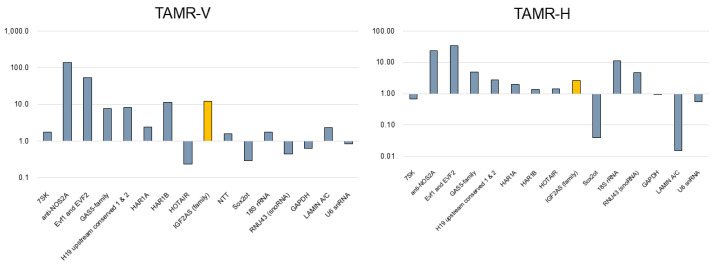
Expression profile of various lncRNAs in tamoxifen-resistant breast cancer cell lines (TAMR-V and TAMR-H) compared to conventional MCF7 cells. IGF2-AS (yellow bar) expression was notably higher in both TAMR cell lines.

IGF2-AS was expressed at higher levels in MCF7 and TNBC than in other cell lines, with the highest expression observed in MCF7 (Figure 1A–C). Additionally, IGF2-AS was highly expressed in both TAMR-V and TAMR-H cells compared to MCF7 breast cancer cells (Figure 2).

### 3.2. Expression of LncRNA IGF2-AS in Breast Cancer Samples Analyzed Using KM Plotter

The genome-wide RNA transcript profile from the KM plotter RNA sequencing (RNA-seq) dataset in breast cancer patients was analyzed. Higher expression of IGF2-AS consistently correlated with poor prognosis. In the overall breast cancer cohort, patients with higher IGF-AS expression had a significantly poorer prognosis in the KM plotter (*p* = 5.3 × 10^−7^). Similarly, in hormone-positive breast cancer and TNBC groups, higher IGF-AS expression was associated with significantly poorer prognosis (*p* = 3.3 × 10^−6^, *p* = 0.01) (Figure 1D–F).

### 3.3. Knockdown of IGF2-AS Reduced Cell Proliferation and Invasion but Did Not Affect Migration in TAMR-V Cell Lines

**Figure 3:**
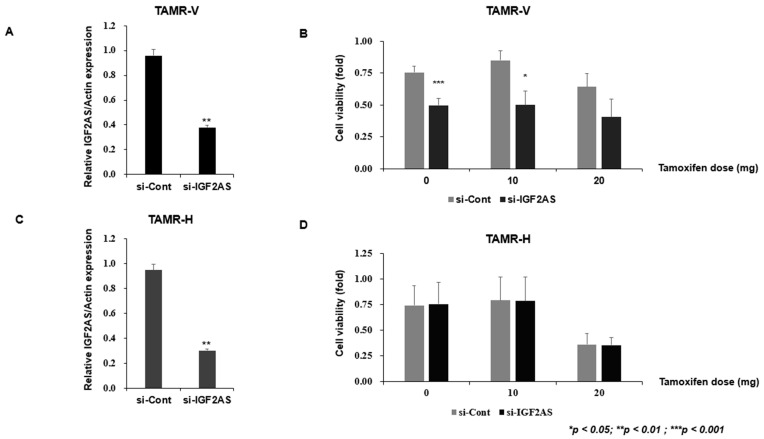
Impact of IGF2-AS knockdown on cell viability in tamoxifen-resistant (TAMR) breast cancer cell lines. Error bars indicate standard deviation (SD) from three independent experiments. (**A**,**C**) Confirmation of IGF2-AS knockdown in TAMR cell lines. Both TAMR breast cancer cell lines were treated with si-IGF2-AS for 48 h, and qRT-PCR was used to analyze IGF2-AS expression. (**B**,**D**) TAMR breast cancer cell lines were treated with si-IGF2-AS for 48 h; cell viability was measured by MTT assay.

To confirm tamoxifen resistance, TAMR-V and TAMR-H cells were treated with tamoxifen or tamoxifen 4-OH. To investigate the function of IGF2-AS in TAMR breast cancer, TAMR-V and TAMR-H cells were transfected with si-IGF2-AS. Both TAMR breast cancer cell lines were treated with si-IGF2-AS for 48 h. IGF2-AS expression was analyzed by qRT-PCR (Figure 3A,C).

The MTT assay was conducted to evaluate the sensitivity and viability changes of TAMR cells according to the tamoxifen dose. The viability of the TAMR-V cells was reduced when the knockdown was applied to the cells, regardless of tamoxifen dose (Figure 3B). In contrast, the viability of the AMR-H cells remained similar between control and knockdown cells regardless of tamoxifen dose. However, the viability of both control and knockdown TAMR-H decreased according to the dose of tamoxifen (Figure 3D).

**Figure 4:**
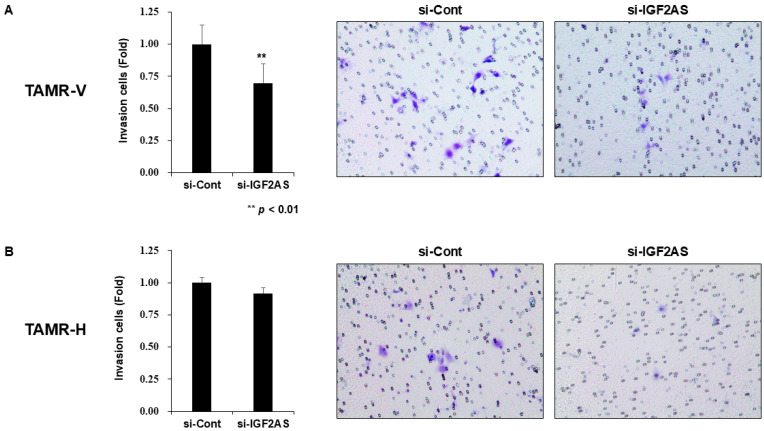
Impact of IGF2-AS knockdown on cell invasion in tamoxifen-resistant (TAMR) breast cancer cell lines. Error bars indicate standard deviation (SD) from three independent experiments. Transwell invasion assay with TAMR-V (**A**) and TAMR-H cell lines (**B**). TAMR breast cancer cell lines were transfected with control siRNA and si-IGF2-AS for 24 h. The transfected cells were harvested and seeded in a Matrigel-coated transwell. After 48 h, the number of invading cells was assessed. The microscope magnification was 200×.

**Figure 5:**
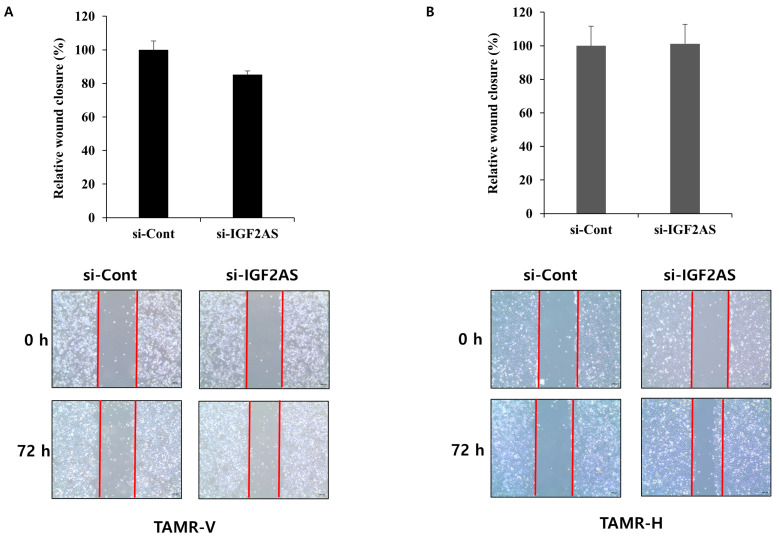
Impact of IGF2-AS knockdown on cell migration in tamoxifen-resistant (TAMR) breast cancer cell lines and the wound healing assay with TAMR-V (**A**) and TAMR-H cell lines (**B**). si-IGF2AS- or control siRNA-transfected TAMR breast cancer cell lines were scratched with a pipette tip, and wound images were captured at 0, 48, and 72 h. The wound area was measured by the ImageJ wound healing tool. Error bars indicate standard deviation (SD) from three independent experiments, and red lines are borderline between cells and scratched wound, which is placed to show clear wound area. The microscope magnification was 40×.

In TAMR-V cells with downregulated IGF2-AS, a significant decrease in cell invasion was observed compared to the control (Figure 4A). However, there were no significant differences in cell invasion between the experimental groups in TAMR-H cells (Figure 4B). Upon examining cell migration through the wound healing assay, no significant differences were observed between the experimental groups in both cells with downregulated IGF2-AS (Figure 5A,B).

### 3.4. Differences Between TAMR-V and TAMR-H Breast Cancer Cells

Although the same experimental processes were conducted, the results from the TAMR-V and TAMR-H cell lines showed some variation. We conducted an analysis using NGS to determine the cause of these varying results among cell lines with the same tamoxifen resistance. Our analysis revealed that gene expression can vary among tamoxifen-resistant cell lines. When analyzing genes that were increased or decreased by more than 2-fold or 4-fold, the gene expression distributions of the two cell lines were very different. Specifically, 54 genes exhibited more than a 2-fold difference, and only three showed more than a 4-fold difference (Figure S1A,B). Scatterplot analysis of gene expression profiling for tamoxifen-resistant and tamoxifen-sensitive breast cancer cell lines also showed different patterns between TAMR-V and TAMR-H cell lines (Figure S2A,B).

We analyzed differentially expressed genes (DEG) using the ExDEGA program. We examined genes in both TAMR cell lines, TAMR-V and TAMR-H, which displayed an increase or decrease in expression compared to MCF-7 cells (Table 2).

### 3.5. Higher Expression of LncRNA IGF2-AS in TAMR Breast Cancer Patients

**Figure 6:**
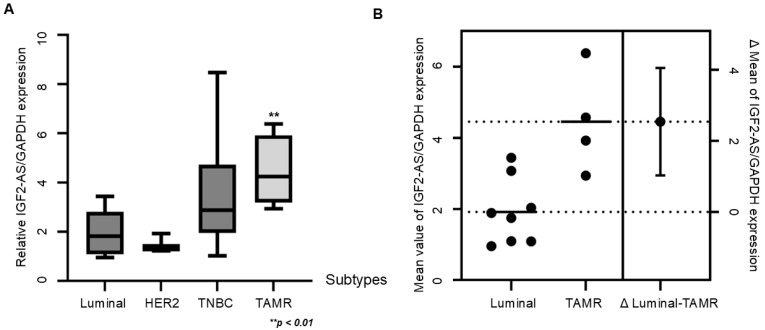
LncRNA IGF2-AS expression was significantly higher in patients with tamoxifen-resistant (TAMR) breast cancer compared to patients with other subtypes. (**A**) The boxplot of gene expression of IGF2-AS compared to GAPDH expression in patients with luminal subtype (*n* = 8), HER2 subtype (*n* = 2), triple-negative subtype (*n* = 8), and TAMR patients (*n* = 4). Error bars indicate standard deviation (SD) from three independent experiments. (**B**) Mean value of IGF2-AS expression in luminal type and TAMR breast cancers and the difference in mean values. The dot lines indicate the average value of each type and error bar indicate margin of error for the average value.

Among various breast cancer subtypes, including luminal (*n* = 8), HER2 (*n* = 2), TNBC (*n* = 8), and TAMR (*n* = 4), the relative expression of lncRNA IGF2-AS/GAPDH was significantly higher in TAMR breast cancer patients compared to patients with other subtypes (*p* < 0.01). Additionally, the difference in mean values between luminal and TAMR breast cancers was greater than 2 (Figure 6A,B).

## 4. Discussion

This study showed that knocking down IGF2-AS reduced cell proliferation and invasion in TAMR-V cells but did not affect migration. However, this knockdown did not impact TAMR-H cells similarly, highlighting differences between these two TAMR cell lines. Tamoxifen is predominantly activated through cytochrome P450-mediated pathways after oral administration, resulting in the formation of several metabolites, including the hydroxylated metabolites 4-hydroxytamoxifen (4-OH-TAM) and 4-hydroxy-*N*-desmethyl-tamoxifen (Endoxifen) [38]. The two TAMR cell lines used in this study differ in that they were generated by tamoxifen and tamoxifen 4-OH treatment. Interestingly, two cell lines were derived from the same source that revealed significant differences in gene expression, as shown by NGS. Although it is unclear which cell line more accurately reflects the human metabolic process, these results highlight that the selection of a specific cell line may impact the outcomes of tamoxifen resistance.

LncRNA IGF2-AS, previously known to be involved only in diabetic retinopathy or angiogenesis in type 3 diabetes mellitus [39,40], has recently been reported to be overexpressed in various malignancies, showing different expressions. While IGF2-AS was downregulated in prostatic cancer [41], it was upregulated in hepatocellular carcinoma, thyroid cancer, and gastric cancer [42,43,44]. Interestingly, while IGF2-AS was upregulated in MCF-7 and T47D breast cancer cell lines [22], it showed higher expression in TAMR cells compared to MCF-7 in this study. Similarly, in the tissue assay, IGF2-AS was significantly highly expressed in tamoxifen-resistant breast cancer compared to other subtypes, including luminal, HER2, and TNBC.

However, the effects of IGF2-AS on proliferation and metastasis-related behaviors varied between the two tamoxifen-resistant breast cancer cell lines. Upon si-IGF2-AS transfection, we observed that IGF2-AS knockdown altered cell viability in TAMR-V cells in a tamoxifen dose-dependent manner, as shown by MTT assays, whereas no significant change was noted in TAMR-H cells. Similarly, Transwell invasion assays revealed a marked reduction in invasive capacity in TAMR-V cells following IGF2-AS knockdown, but this effect was absent in TAMR-H cells. Interestingly, cell migration—assessed by wound healing assays—was unaffected in both cell lines, indicating that IGF2-AS may be more closely associated with invasion than migration.

These findings suggest that the biological role of IGF2-AS in tamoxifen resistance is not uniform across all resistant models. The discrepancy between TAMR-V and TAMR-H responses highlights the complexity and heterogeneity of endocrine resistance mechanisms in breast cancer. While IGF2-AS may not function as a universal biomarker for all tamoxifen-resistant subtypes, it appears to play a critical role in specific contexts. This subtype-specific dependency underscores the potential of IGF2-AS as a precision biomarker, suggesting a rationale for its use in stratified therapeutic approaches rather than broad, non-specific applications.

Resistance to tamoxifen has been partly attributed to increased expression of ATP-binding cassette (ABC) transporters, which mediate drug efflux and reduce intracellular drug accumulation. Among these, breast cancer resistance protein (BCRP) has been frequently related to endocrine therapy resistance in breast cancer. Given the role of lncRNAs in regulating drug resistance pathways, IGF2-AS may influence tamoxifen sensitivity by modulating the expression of ABC transporters. Further studies are necessary to determine whether IGF2-AS knockdown leads to decreased BCRP expression, which could offer novel insights into its functional role in mediating endocrine resistance.

Several genes were commonly highly expressed in TAMR-V and TAMR-H cells compared to MCF-7 cells, including LGALS3, EPAS1, BCAS1, ADM, Keratin-80 (KRT80), C19orf33, UPK2, ANXA3, ANXA9, TNS3, SYTL1, CXXC5, and ST3GAL4. Among these, EPAS1 is associated with paclitaxel-resistant breast cancer, and KRT80 was recently reported to have an association with endocrine-resistant ERα breast cancer [45,46]. Additionally, ANXA3 is related to drug resistance in breast cancer [47]. The expression of these genes was commonly higher in TAMR-V and TAMR-H cells, regardless of metabolites, which means they may serve as potential targets for therapeutic development.

One of the major limitations of this study is the exclusive reliance on in vitro experiments. While the upregulation of IGF2-AS was confirmed in TAMR tissue samples, the precise mechanisms were not clearly demonstrated. Moreover, the two TAMR cell lines used in the study exhibited different outcomes, making it challenging to determine which cell line most closely mirrors human metabolic processes. To strengthen the translational relevance of the findings, future studies should incorporate in vivo validation approaches, such as xenograft models or breast cancer organoid systems.

Despite the limitations in cell models and variability, the study establishes the biological role of IGF2-AS and identifies common genes as potential therapeutic targets. However, considering the different roles that IGF2-AS appears to play in tamoxifen-resistant breast cancer cells, future research should investigate how IGF2-AS contributes to hormone therapy resistance, especially in specific subtypes of breast cancer. To confirm its clinical importance, studies using patient-derived models such as xenografts, organoids, or actual tumor tissues will be required. It will also be important to test whether blocking IGF2-AS works better when combined with existing hormone therapies like selective estrogen receptor modulators (SERMs) or CDK4/6 inhibitors. These combination treatments might help overcome resistance in patients who do not respond well to standard therapy alone.

## 5. Conclusions

This study demonstrates that IGF2-AS is selectively upregulated in tamoxifen-resistant breast cancer and promotes cell proliferation and invasion in a cell line-specific manner, supporting its role as a key factor and potential therapeutic target. The observed differences in cell viability and invasion reflect the complexity of resistance mechanisms and the need for personalized treatment strategies. Also, NGS revealed distinct gene expression profiles across resistant cell lines, emphasizing the multifaceted nature of endocrine resistance. Despite inherent limitations in in vitro models, this study establishes the biological relevance of IGF2-AS and identifies commonly altered genes as additional therapeutic candidates. Future research will aim to validate these findings in patient-derived models and assess the clinical utility of targeting IGF2-AS to overcome endocrine resistance.

## Figures and Tables

**Table 1:** Expression level of long non-coding RNAs (lncRNAs) in tamoxifen-resistant breast cancer cell lines (TAMR-V and TAMR-H).

Level of Expression	lncRNAs	TAMR-V *	TAMR-H *
Upregulation	Anti-*NOS2A*	52.82983 ± 16.65489	24.41382 ± 9.56836
	*EVF1* and *EVF2*	34.11834 ± 12.70678	28.66012 ± 10.95019
	*HAR1B*	9.013918 ± 8.091384	4.250339 ± 4.595892
	*IGF2AS* (family)	4.865702 ± 2.040343	1.772124 ± 0.980745
	*GAS5* (family)	3.377255 ± 0.835037	4.224700 ± 0.311261
	*H19* upstream conserved 1 & 2	3.027784 ± 1.649675	2.038056 ± 0.510124
Downregulation	*NTT*	0.66975 ± 0.3869	0.767579 ± 0.308688
	*7SK*	0.656233 ± 0.028282	0.682445 ± 0.061467
	*SOX2OT*	0.094758 ± 0.046917	0.044528 ± 0.023905
Control	18S rRNA	8.822375 ± 0.791897	11.43136 ± 1.015886
	*RNU43* (snoRNA)	1.664141 ± 0.222279	4.614409 ± 0.336516
	*LAMIN A/C*	0.547854 ± 0.02251	0.016867 ± 0.009027
	*U6* snRNA	0.31399 ± 0.060132	0.558524 ± 0.121545
	*GAPDH*	0.271698 ± 0.027552	0.983259 ± 0.12848

* Values are presented as mean ± SD.

**Table 2:** Characteristics of major genes and expression levels in tamoxifen-resistant breast cancer cells (TAMR-V and TAMR-H) compared to MCF7.

Gene Symbol	TAMR_V/MCF7	TAMR_H/MCF7	Transcript ID	Biotype	Aliases	Description	NCBI Search
LGALS3	19.198	267.620	NM_001177388	protein_coding	CBP35|GAL3|GALBP|GALIG|L31|LGALS2|MAC2	Lectin, galactoside-binding soluble 3	LGALS3
EPAS1	18.378	70.987	NM_001430	protein_coding	ECYT4|HIF2A|HLF|MOP2|PASD2|bHLHe73	Endothelial PAS domain protein 1	EPAS1
BCAS1	9.121	16.782	NM_003657	protein_coding	AIBC1|NABC1	Breast carcinoma amplified sequence 1	BCAS1
ADM	8.136	7.956	NM_001124	protein_coding	AM	Adrenomedullin	ADM
KRT80	7.849	6.289	NM_182507	protein_coding	KB20	Keratin 80	KRT80
C19orf33	7.505	36.531	NM_033520	protein_coding	H2RSP|IMUP|IMUP-1|IMUP-2|MGC39135|MGC75180	Chromosome 19 open reading frame 33	C19orf33
UPK2	7.335	83.240	NM_006760	protein_coding	MGC138598|UP2|UPII	Uroplakin 2	UPK2
ANXA3	7.235	7.402	NM_005139	protein_coding	ANX3	Annexin A3	ANXA3
TNS3	7.102	5.821	NM_022748	protein_coding	DKFZp686K12123|DKFZp686M1045|FLJ13732|FLJ35545|H_NH049I23.2|MGC88434|TEM6|TENS1	Tensin 3	TNS3
ANXA9	6.39	12.706	NM_003568	protein_coding	ANX31	Annexin A9	ANXA9
SYTL1	5.364	5.411	NM_001193308	protein_coding	FLJ14996|JFC1|SLP1	Synaptotagmin like 1	SYTL1
CXXC5	5.096	7.979	NM_016463	protein_coding	CF5|RINF|WID	CXXC finger protein 5	CXXC5
ST3GAL4	5.038	10.386	NM_001254757	protein_coding	CGS23|FLJ11867|FLJ46764|NANTA3|SAT3|SIAT4|SIAT4C|ST3GalIV|STZ	ST3 beta-galactoside alpha-2,3-sialyltransferase 4	ST3GAL4
S100A4	0.190	0.126	NM_019554	protein_coding	18A2|42A|CAPL|FSP1|MTS1|P9KA|PEL98	S100 calcium-binding protein A4	S100A4
CXCL12	0.159	0.040	NM_001277990	protein_coding	IRH|PBSF|SCYB12|SDF1|SDF1A|SDF1B|TLSF|TPAR1	C-X-C motif chemokine ligand 12	CXCL12
MUCL1	0.126	8.281	NM_058173	protein_coding	SBEM	Mucin-like 1	MUCL1
TEX19	0.124	0.114	NM_207459	protein_coding	FLJ35767	Testis expressed 19	TEX19
FHL1	0.069	0.096	NR_027621	protein_coding	FHL1A|FHL1B|FLH1A|KYOT|MGC111107|SLIM1|SLIMMER|XMPMA	Four and a half LIM domains 1	FHL1

## Data Availability

The datasets generated and/or analyzed during the current study are not publicly available. However, they are available from the corresponding author upon reasonable request.

## Supplementary Materials

The following supporting information can be downloaded at: https://www.mdpi.com/article/10.3390/biomedicines13092087/s1, Figure S1: Venn diagram of gene expression profiles between tamoxifen-resistant breast cancer cell lines (TAMR-V, TAMR-H) and MCF7 cells based on next-generation sequencing analysis; Figure S2: Scatterplot analysis of gene expression profiling for tamoxifen-resistant breast cancer cells (TAMR-V [A]; TAMR-H [B]) and MCF7 breast cancer cell lines.

## Abbreviations

The following abbreviations are used in this manuscript:

LncRNAs	Long non-coding RNAs
IGF2-AS	Insulin-like growth factor 2 antisense
TAMR	Tamoxifen resistance
NGS	Next-generation sequencing
ER	Estrogen receptor
PR	Progesterone receptor
HER2	Human epidermal growth factor receptor 2
TNBC	Triple-negative breast cancer
SERMs	Selective estrogen receptor modulators
OS	Overall survival
