# Supplementary material for: Differential Expression of Long Non-Coding RNA IGF2-AS in Tamoxifen-Resistant Breast Cancer Cells
Source: Biomedicines. 2025 Aug 27;13(9):2087. doi: 10.3390/biomedicines13092087 (PMC12467862; doi:10.3390/biomedicines13092087)
Supplement: Supplementary file 1 [file biomedicines-13-02087-s001.zip › biomedicines-3772518-supplementary.pdf]

biomedicines

Supplementary information

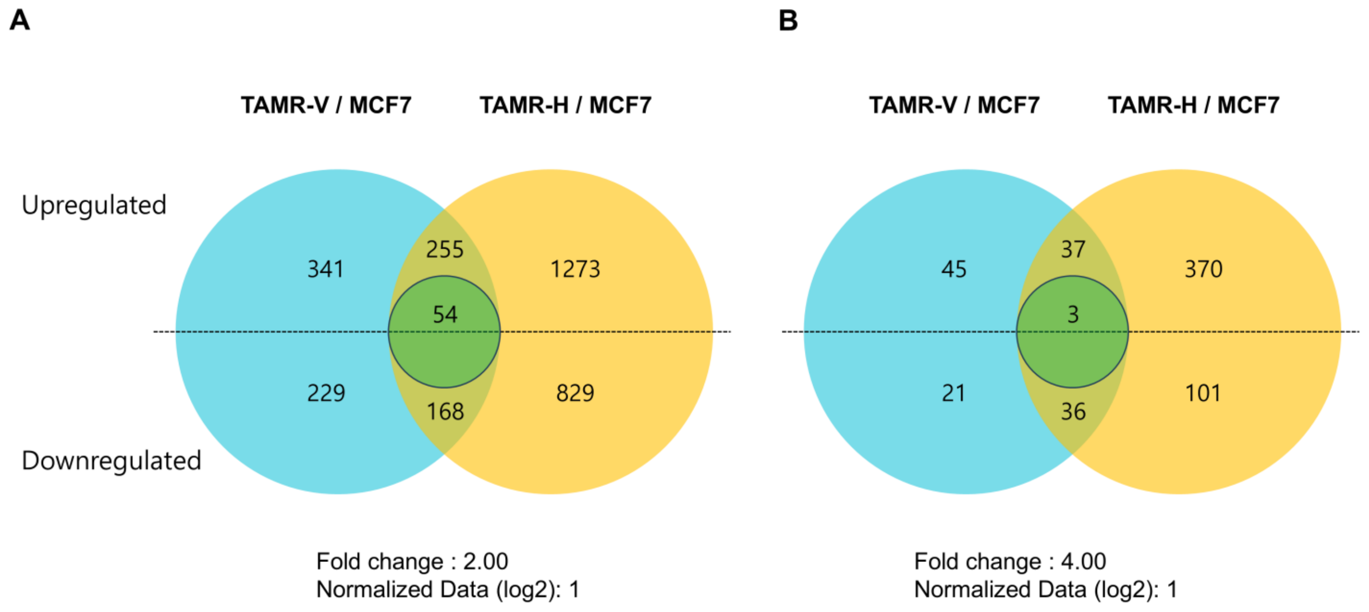

Figure S1. Venn diagram of gene expression profiles between tamoxifen-resistant breast cancer cell lines (TAMR-V, TAMR-H) and MCF7 cells based on next-generation sequencing analysis. The blue circles indicate genes that are increased or decreased in TAMR-V compared to MCF-7 cells, and the yellow circles indicate genes that are increased or decreased in TAMR-H compared to MCF-7 cells. Green circles represent the number of cells in which gene expression was up or down in opposite directions. (**A**) Genes differentially expressed at levels greater than 2-fold between tamoxifen-resistant and MCF-7 breast cancer cell lines. (**B**) Genes differentially expressed at levels greater than 4-fold between tamoxifen-resistant and MCF-7 breast cancer cell lines.

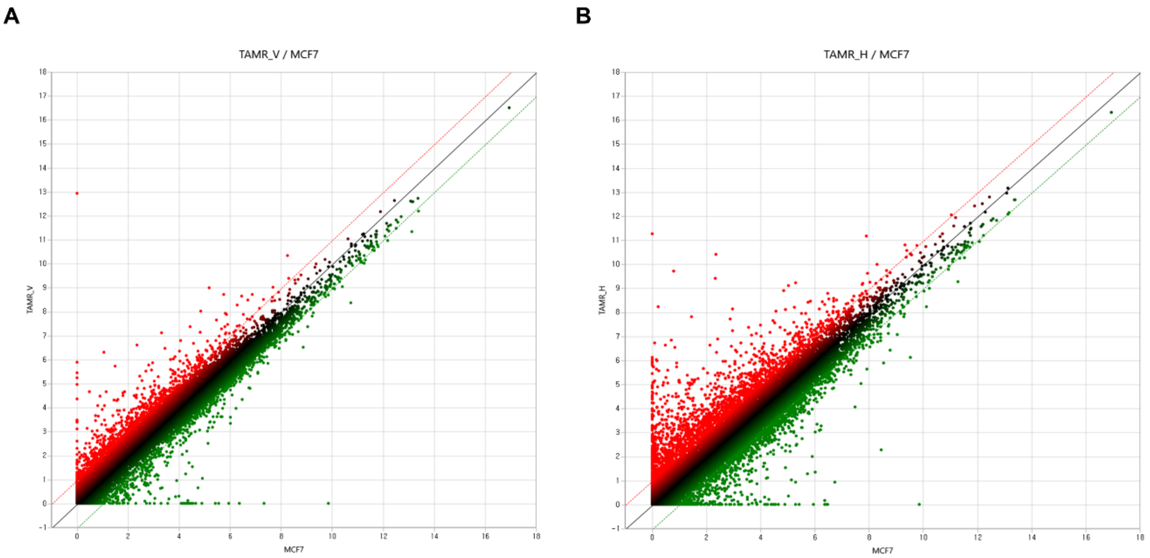

Figure S2. Scatterplot analysis of gene expression profiling for tamoxifen-resistant breast cancer cells (TAMR-V [**A**], TAMR-H [**B**]), and MCF7 breast cancer cell lines. The scatterplot analysis reveals different patterns between the two cell lines. The red line indicates the cutoff for ≥2-fold upregulation, and ≥2-fold downregulation is indicated by the green line.
